# Applications of Bayesian Gene Selection and Classification with Mixtures of Generalized Singular ***g***-Priors

**DOI:** 10.1155/2013/420412

**Published:** 2013-12-08

**Authors:** Wen-Kuei Chien, Chuhsing Kate Hsiao

**Affiliations:** ^1^Biostatistics Center, Taipei Medical University, Taipei 11031, Taiwan; ^2^Institute of Epidemiology and Preventive Medicine, College of Public Health, National Taiwan University, Taipei 10055, Taiwan; ^3^Bioinformatics and Biostatistics Core, Division of Genomic Medicine, Research Center for Medical Excellence, National Taiwan University, Taipei 10055, Taiwan

## Abstract

Recent advancement in microarray technologies has led to a collection of an enormous number of genetic markers in disease association studies, and yet scientists are interested in selecting a smaller set of genes to explore the relation between genes and disease. Current approaches either adopt a single marker test which ignores the possible interaction among genes or consider a multistage procedure that reduces the large size of genes before evaluation of the association. Among the latter, Bayesian analysis can further accommodate the correlation between genes through the specification of a multivariate prior distribution and estimate the probabilities of association through latent variables. The covariance matrix, however, depends on an unknown parameter. In this research, we suggested a reference hyperprior distribution for such uncertainty, outlined the implementation of its computation, and illustrated this fully Bayesian approach with a colon and leukemia cancer study. Comparison with other existing methods was also conducted. The classification accuracy of our proposed model is higher with a smaller set of selected genes. The results not only replicated findings in several earlier studies, but also provided the strength of association with posterior probabilities.

## 1. Introduction 

Recent advancement in oligonucleotide microarray technologies has resulted in production of thousands of gene expression levels in a single experiment. With such vast amount of data, one major task for researchers is to develop classification rules for prediction of cancers or cancer subtypes based on gene expression levels of tissue samples. The accuracy of such classification rules may be crucial for diagnosis and treatment, since different cancer subtypes may require different target-specific therapies. However, the development of good and efficient classification rules has not been straightforward, either because of the huge number of genes collected from a relatively small number of tissue samples or because of the model complexity associated with the biological mechanism. The identification of a smaller set of relevant genes to characterize different disease classes, therefore, has been a challenging task. Procedures which are efficient in gene selection as well as in classification do play an important role in cancer research.

Many approaches have been proposed for classes classification. For example, several analyses identified a subset of classifying genes with *t*-statistics, regression model approach, mixture model, Wilcoxon score test, or the between-within classes sum of squares (BSS/WSS) [1–7]. These methods are univariate in the sense that each gene is tested individually. Others started with an initial step of dimension reduction before classification procedures, such as the principle components analysis (PCA) [8–10] and the partial least squares algorithm (PLS algorithm) [11–15]. These methods may reduce dimension (the number of genes) effectively but may not be biologically interpretable. To capture the gene-gene correlations, researchers proposed the pair-based method [16], correlation-based feature selection [17], and the Markov random field prior [18]. Although these methods can model the gene-gene interaction, they can be computationally time-consuming.

Bayesian approach can accommodate naturally the interplay between genes via prior distributions, under the setting of regression models. Examples included the Bayesian hierarchical mixture model [19–21] and a logistic or probit link with latent variables and stochastic search variable selection (SSVS) procedure for binary and multicategorical phenotypes [22–25]. To consider all genes simultaneously, most Bayesian approaches adopt a multivariate analysis with a natural conjugate prior *N*(0, *c*(**X** ^*T*^ **X**)^−1^), called *g*-prior, for the regression parameters **β** [26]. This *a priori* distribution utilizes the design matrix as the prior covariance matrix of **β** and can lead to a relatively simple posterior distribution. However, if the number of genes is much larger than the number of samples available, the dimension of **X** becomes large and a high degree of multicollinearity may occur. In that case, the covariance matrix of Zellner's *g*-prior becomes nearly singular. Modifications included the *gsg*-prior distribution with the Moore-Penrose generalized inverse matrix [27] and use of a ridge parameter [28, 29]. Alternatively, other researchers focused on the scalar *c* in *c*(**X** ^*T*^ **X**)^−1^ which controls the expected size of the nonzero regression coefficients. For instance, it was reported that the final results are insensitive to the values of *c* between 10 and 100, and the value *c* = 100 has been suggested after extensive examinations [30]. Instead of fixing *c* at a constant, George and Foster [31] proposed an empirical Bayes estimate for *c*, while Liang and colleagues [32] suggested a hyper-*g* prior, a special case of the incomplete inverse-gamma prior in Cui and George [33].

The main purpose of this research is the application of fully Bayesian approaches with a hyperprior on *c*. Specifically we adopted an inverse-gamma prior IG(1/2, *n*/2) which was commented earlier that it could lead to computational difficulty. Therefore, we outlined a MCMC algorithm and demonstrated its implementation. In this paper, we considered a probit regression model for classification with SSVS to identify the influential genes, augmented the response variables *Y*_1_, *Y*_2_,…, *Y*_*n*_ with latent variables *Z*_1_, *Z*_2_,…, *Z*_*n*_, and converted the probit model to a Gaussian regression problem with the generalized singular *g*-prior (*gsg*-prior). For the choice of *c*, we assigned a hyperprior for the uncertainty in *c*. This hyperprior is intuitive and differs from those in [32, 33]. Finally, we defined an indicator variable *γ*_*j*_ for the *j*th gene and perform MCMC methods to generate posterior samples for gene selection and class classification. The rest of the paper is arranged as follows. In Section 2, we briefly described the model specification including the data augmentation approach and SSVS methods. Under this hyperprior on *c*, we also demonstrated the implementation of the Bayesian inference. Applications of three cancer studies, acute leukemia, colon cancer, and large B-cell lymphoma (DLBCL), were presented in Section 3. Conclusion and discussion were given in Section 4.

## 2. Model and Notation 

Let (**X**, **Y**) indicate the observed data,

(1) $$\begin{array}{c} X=\left(\begin{array}{cccc} x_{11} & x_{12} & \cdots & x_{1p}\\ x_{21} & x_{22} & \cdots & x_{2p}\\ \vdots & \vdots & \ddots & \vdots \\ x_{n1} & x_{n2} & \cdots & x_{np} \end{array}\right), \end{array}$$

where *x*_*ij*_ denotes the expression level of the *j*th gene from the *i*th sample and **Y** = (*Y*_1_, *Y*_2_,…, *Y*_*n*_)^*T*^ denotes the response vector, where *Y*_*i*_ = 1 indicates that sample *i* is a cancer tissue and *Y*_*i*_ = 0 for normal tissue. Assume that *Y*_1_, *Y*_2_,…, *Y*_*n*_ are *n* independent random variables with *p*_*i*_ = Pr(*Y*_*i*_ = 1).

### 2.1. Probit Model with Latent Variable

The gene expression measurements can be linked to the response outcome with a probit regression model:

(2) $$\begin{array}{c} p_{i}=\text{Pr}\left(Y_{i}=1\right)=\Phi \left(\alpha +X_{i}\beta \right), \end{array}$$

where *α* represents the intercept, **X**_*i*_ is the *i*th row in the *n* × *p* design matrix **X**, **β** = (*β*_1_,…, *β*_*p*_)^*T*^ is the vector of regression coefficients, and Φ is the standard normal cumulative distribution function.

To perform statistical inference under this probit regression model, we first adopt *n* independent latent variables *Z*_1_, *Z*_2_,…, *Z*_*n*_, where

(3) $$\begin{array}{c} Z_{i}=\alpha +X_{i}\beta +\varepsilon_{i},\;\varepsilon_{i}\sim N\left(0,1\right),\;\;i=1,\ldots ,n, \end{array}$$

and the *Z*_*i*_ corresponds to the disease status as

(4) $$\begin{array}{c} Y_{i}=\{\begin{array}{cc} 1, & \text{if}\;\;Z_{i}>0,\\ 0, & \text{if}\;\;Z_{i}\leq 0. \end{array} \end{array}$$

The use of such latent variables helps to determine which category the *i*th sample is to be classified. Note that multiplying a constant on both sides in (3) does not change the model; thus a unit variance is considered for *ε*_*i*_.

If a noninformative prior is assumed for **β**, then the posterior covariance matrix of **β** given **Z** ≡ (*Z*_1_, *Z*_2_,…, *Z*_*n*_) becomes (**X** ^*T*^ **X**)^−1^. However, due to the enormous size of microarray data, (**X** ^*T*^ **X**)^−1^ may be nearly singular, and variable selection for dimension reduction is needed. We define for variable selection the vector **γ** ≡ (*γ*_1_, *γ*_2_,…, *γ*_*p*_) whose elements are all binary, where

(5) $$\begin{array}{c} \gamma_{i}=\{\begin{array}{cc} 1, & \text{if}\;\;\beta_{i}\neq 0\;\left(\text{the}\;\;i\text{th}\;\;\text{gene}\;\;\text{selected}\right),\\ 0, & \text{if}\;\;\beta_{i}=0\;\left(\text{the}\;\;i\text{th}\;\;\text{gene}\;\;\text{not}\;\;\text{selected}\right). \end{array} \end{array}$$

Given **γ**, we denote *p* ^*γ*^ as the number of 1's in **γ** and **β** ^*γ*^ a *p* ^*γ*^ × 1 reduced vector containing the regression coefficients *β*_*j*_ if its corresponding *γ*_*j*_ is 1. Accordingly, for all *γ*_*j*_ = 1, the corresponding columns in **X** are collected to build **X** ^*γ*^, an *n* × *p* ^*γ*^ reduced gene expression matrix. Given **γ**, the probit regression model in (3) can be written as

(6) $$\begin{array}{c} Z_{i}=\alpha +X_{i}^{\gamma }\beta^{\gamma }+\varepsilon_{i},\;\varepsilon_{i}\sim N\left(0,1\right),\;\;i=1,\ldots ,n, \end{array}$$

where **X**_*i*_^*γ*^ is the *i*th row in **X** ^*γ*^.

### 2.2. Choice of Prior Distributions

To complete the model specification, we assign a normal *N*(0, *h*) prior for the intercept *α* with a large *h* indicating no *a priori* information. For the regression parameters, the commonly applied *g*-prior **β** ^*γ*^ | **γ**, *c* ~ *N*(0, *c*(**X** ^*γ*^ ^*T*^ **X** ^*γ*^)^−1^) may not work if the sample size *n* is less than the number *p* ^*γ*^, leading to the results that **X** ^*γ*^ ^*T*^ **X** ^*γ*^ is not of full rank and (**X** ^*γ*^ ^*T*^ **X** ^*γ*^)^−1^ does not exist. Therefore, we consider the *gsg*-prior distribution with (**X** ^*γ*^ ^*T*^ **X** ^*γ*^)^+^ as the pseudoinverse of **X** ^*γ*^ ^*T*^ **X** ^*γ*^ for **β** ^*γ*^ conditioning on (**γ**, *c*), **β** ^*γ*^ | **γ**, *c* ~ *N*(0, *c*(**X** ^*γ*^ ^*T*^ **X** ^*γ*^)^+^). This would solve the singularity problem. Next, we assign for **γ** and *c* the priors

(7) $$\begin{array}{c} \pi \left(c\right)=\frac{{\left(n/2\right)}^{1/2}}{\Gamma \left(1/2\right)}c^{-3/2}e^{-n/(2c)},\\ \gamma_{i}\sim \text{Ber}\left(\pi_{i}\right),\;0\leq \pi_{i}\leq 1,\;\;i=1,\ldots ,p, \end{array}$$

and assume that *γ*_*i*_ are independent for *i* = 1,…, *p*. Note that here the *π*_*i*_'s are of small values, implying a small set of influential genes.

We now complete the model specification:

(8) Y=(Y1,Y2,…,Yn)T, wherepi=Pr(Yi=1)=Φ(α+Xiβ),Zi=α+Xiγβγ+εi, whereYi=1  if  Zi>0,  and  0  otherwiseβγ ∣ γ,c~N(0,c(XγTXγ)+),π(c)~IG(12,n2),γi~Ber(πi).

Note that *Y*_*i*_ = 1 if the *i*th sample is a cancer tissue, *α* is the intercept, **β** = (*β*_1_,…, *β*_*p*_)^*T*^ is the vector of regression coefficients, Φ is the standard normal cumulative distribution function, and **X** is the design matrix:

(9) $$\begin{array}{c} X=\left(\begin{array}{cccc} x_{11} & x_{12} & \cdots & x_{1p}\\ x_{21} & x_{22} & \cdots & x_{2p}\\ \vdots & \vdots & \ddots & \vdots \\ x_{n1} & x_{n2} & \cdots & x_{np} \end{array}\right). \end{array}$$

And **γ** ≡ (*γ*_1_, *γ*_2_,…, *γ*_*p*_) contains the binary *γ*_*i*_, where *γ*_*i*_ = 1 if the *i*th gene is selected (*β*_*i*_ ≠ 0), **β** ^*γ*^ is a *p* ^*γ*^ × 1 reduced vector containing the regression coefficients *β*_*j*_ if its corresponding *γ*_*j*_ is 1, *p* ^*γ*^ is the number of 1's in **γ**, and **X**_*i*_^*γ*^ is the *i*th row in **X** ^*γ*^.

### 2.3. Computation and Posterior Inference

Based on the prior distributions specified in previous sections, the joint posterior distribution can be derived as

(10) P(Z,α,βγ,γ,c ∣ Y,X)  ∝[exp⁡{−∑i=1n(Zi−α−Xiγβγ)22}∏i=1nI(Ai)]   ·exp⁡⁡(−α22h)   ·[exp⁡⁡(−βγTXγTXγβγ2c)∏i=1mγλi−1/2]   ·[∏i=1pπiγi(1−πi)1−γi]   ·[c−3/2exp⁡(−n2c)],

where

(11) $$\begin{array}{c} A_{i}=\{\begin{array}{cc} \left\{Z_{i}:Z_{i}>0\right\} & \text{if}\;\;Y_{i}=1,\\ \left\{Z_{i}:Z_{i}\leq 0\right\} & \text{if}\;\;Y_{i}=0, \end{array} \end{array}$$

and *λ*_1_, *λ*_2_,…, *λ*_*m*_**γ**__  (*m*_**γ**_ ≤ *p*_**γ**_) are the nonzero eigenvalues of (**X** ^*γ*^ ^*T*^ **X** ^*γ*^)^+^. From (10), **β** ^*γ*^ given (**Z**, *α*, **γ**, *c*, **Y**, **X**) is a multivariate normal distribution with a covariance matrix *c*(**X** ^*γ*^ ^*T*^ **X** ^*γ*^)^+^/(*c* + 1). In the case where **X** ^*γ*^ is not of full column rank, the problem of convergence may occur in the MCMC algorithm because the covariance matrix is not positive definite and the multivariate normal distribution becomes degenerated. To avoid this problem and speed up the computations, we integrate out *α* and **β** ^*γ*^ in (10) following Yang and Song's [27] suggestion and derive

(12) p(Z,γ,c ∣ Y,X)  ∝1|Σγ|1/2exp⁡(−ZTΣγ−1Z2)∏i=1nI(Ai)   ·∏i=1pπiγi(1−πi)1−γic−3/2e−n/2c,

where Σ_*γ*_ = **I**_*n*_ + *h*11^*T*^ + *c* **X** ^*γ*^(**X** ^*γ*^ ^*T*^ **X** ^*γ*^)^+^ **X** ^*γ*^ ^*T*^. As the posterior distribution is not available in an explicit form, we use the MCMC technique to obtain posterior sample observations. The computational sampling scheme is as follows.

(1) Draw **Z** from *p*(**Z** | **Y**, **X**, **γ**, *c*), where
  (13) $$\begin{array}{c} p\left(Z\;|\;Y,X,\gamma ,c\right)\propto N\left(0,\Sigma_{\gamma }\right)\overset{n}{\underset{i=1}{\prod }}I\left(A_{i}\right). \end{array}$$
  The conditional distribution of **Z** given (**Y**, **X**, **γ**, *c*) is a multivariate truncated normal. Since it is difficult to directly sample **Z** from this distribution, we draw samples *Z*_*i*_, *i* = 1,…, *n*, from *p*(*Z*_*i*_ | **Z**_(−*i*)_, **Y**, **X**, **γ**, *c*), where **Z**_(−*i*)_ is the vector of **Z** without the *i*th element [34].
(2) Draw **γ** from *p*(**γ** | **Y**, **X**, **Z**, *c*), where
  (14) p(γ ∣ Y,X,Z,c) ∝1|Σγ|1/2exp⁡(−ZTΣγ−1Z2)∏i=1pπiγi(1−πi)1−γi.
  Similar to the above procedure, we draw samples *γ*_*i*_, *i* = 1,…, *n*, from *p*(*γ*_*i*_ | **γ**_(−*i*)_, **Y**, **X**, **Z**, *c*). It can be shown that
  (15) p(γi ∣ γ(−i),Y,X,Z,c) =p(γi=1 ∣ γ(−i),Y,X,Z,c)p(γi=1 ∣ γ(−i),Y,X,Z,c)+p(γi=0 ∣ γ(−i),Y,X,Z,c) =(1+1−πiπiρ)−1,
  where
  (16) $$\begin{array}{c} \rho ={\left|\Sigma_{\gamma^{1}}\Sigma_{\gamma^{0}}^{-1}\right|}^{1/2}\exp\left\{\frac{Z^{T}\left(\Sigma_{\gamma^{1}}^{-1}-\Sigma_{\gamma^{0}}^{-1}\right)Z}{2}\right\},\\ \gamma^{1}=\left(\gamma_{1},\ldots ,\gamma_{i-1},\gamma_{i}=1,\gamma_{i+1},\ldots ,\gamma_{p}\right),\\ \gamma^{0}=\left(\gamma_{1},\ldots ,\gamma_{i-1},\gamma_{i}=0,\gamma_{i+1},\ldots ,\gamma_{p}\right), \end{array}$$
  Σ_**γ**^1^_ and Σ_**γ**^0^_ are similar to Σ_**γ**_ with **γ** replaced by **γ** ^1^ and **γ** ^0^, respectively.
(3) Draw *c* from *p*(*c* | **Y**, **X**, **Z**, **γ**), where
  (17) p(c ∣ Y,X,Z,γ)  ∝p(Z,γ,c ∣ Y,X)  ∝1|Σγ|1/2exp⁡(−ZΣγ−1Z2)·c−3/2e−n/2c.
  The above distribution does not belong to any standard distribution, so we will use Metropolis-Hastings algorithm to sample *c*.

The iteration therefore starts with initial values of **Z** ^(0)^, **γ** ^(0)^, and *c* ^(0)^, and our MCMC procedures at the *t*th iteration are as follows.

**Step 1**

Draw *Z*_*i*_^(*t*)^ from *p*(*Z*_*i*_ | **Z**_(−*i*)_^(*t*−1)^, **Y**, **X**, **γ** ^(*t*−1)^, *c* ^(*t*−1)^), *i* = 1,…, *n*.

**Step 2**

For *i* = 1,…, *p*, calculate *p*_*i*_^(*t*)^ ≡ *p*(*γ*_*i*_^(*t*)^ = 1 | **γ**_(−*i*)_^(*t*−1)^, **Y**, **X**, **Z** ^(*t*)^, *c* ^(*t*−1)^), generate a random number *u*_*i*_ from *U*(0,1), and let

(18) $$\begin{array}{c} \gamma_{i}^{\left(t\right)}=\{\begin{array}{cc} 1, & u_{i}<p_{i}^{\left(t\right)},\\ 0, & \text{otherwise}. \end{array} \end{array}$$

**Step 3**

Draw *c* from (17) by the following steps:

(i) maximize (17) to obtain *c*_opt_;
(ii) generate the proposal value
  (19) $$\begin{array}{c} c^{\left(t\right)}=c_{\text{opt}}+\varepsilon^{\left(t\right)}, \end{array}$$
  where *ε* ^(*t*)^ follows a normal *N*(*μ*, *σ* ^2^) truncated in a positive region (a,b) with a density *q*;
(iii) accept *c* ^(*t*)^ with the acceptance probability:
  (20) $$\begin{array}{c} R=\min\left\{1,\frac{p\left(c^{\left(t\right)}\;|\;Y,X,Z,\gamma \right)}{p\left(c^{\left(t-1\right)}\;|\;|Y,X,Z,\gamma \right)}\cdot \frac{q\left(c^{\left(t-1\right)}-c_{\text{opt}}\right)}{q\left(c^{\left(t\right)}-c_{\text{opt}}\right)}\right\}. \end{array}$$

After the initial burn-in period, we obtain the MCMC samples {(**Z** ^(*t*)^, **γ** ^(*t*)^, *c* ^(*t*)^), *t* = 1,…, *M*} which are next used to estimate the posterior gene inclusion probability by

(21) $$\begin{array}{c} \hat{p}\left(\gamma_{i}=1\;|\;Y,X\right)=\frac{1}{M}\overset{M}{\underset{t=1}{\sum }}I\left(\gamma_{i}^{\left(t\right)}=1\right), \end{array}$$

and genes with higher posterior inclusion probabilities are considered more relevant to classification.

### 2.4. Classification

To assess the performance of our procedures, testing data sets are considered. For example, a testing set (*X*_new_, *Y*_new_) is available, and the predictive probability of *Y*_new_ given *X*_new_ is

(22) p(Ynew ∣ Y,X,Xnew) =∫p(Ynew ∣ Y,X,Xnew,Z,γ,c)p(Z,γ,c ∣ Y,X)d(Z,γ,c).

Based on the MCMC samples, we estimate the probability with

(23) p^(Ynew ∣ Y,X,Xnew) =1M∑t=1Mp(Ynew ∣ Y,X,Xnew,Z(t),γ(t),c(t)).

When there are no testing sets available, we adopt the leave-one-out cross-validation (LOOCV) method to evaluate the performance with the training data. Because the predictive probability for *Y*_*i*_ is

(24) p(Yi ∣ Y(−i),X)  =(∭p(Yi ∣ Y(−i),X,Z,γ,c)−1     ×p(Z,γ,c ∣ Y,X)dZdγdc)−1,

where **Y**_(−*i*)_ denotes the vector of **Y** without the *i*th element. We estimate this probability based on the generated MCMC samples,

(25) $$\begin{array}{c} \hat{p}\left(Y_{i}\;|\;Y_{\left(-i\right)},X\right)=\frac{M}{\sum_{t=1}^{M}p{\left(Y_{i}\;|\;Y_{\left(-i\right)},X,Z^{\left(t\right)},\gamma^{\left(t\right)},c^{\left(t\right)}\right)}^{-1}}. \end{array}$$

## 3. Applications

In this section, we applied the fully Bayesian approach and the reference prior to three cancer studies: colon cancer, leukemia, and a large B-cell lymphoma (DLBCL) study [35–37]. We also compared the performance of this approach with other existing gene selection and classification methods. These data have been extensively studied with various methods but we only included a limited set of them. Others can be found in the reference lists of the work cited here.

### 3.1. Colon Cancer Study

**Figure 1:**
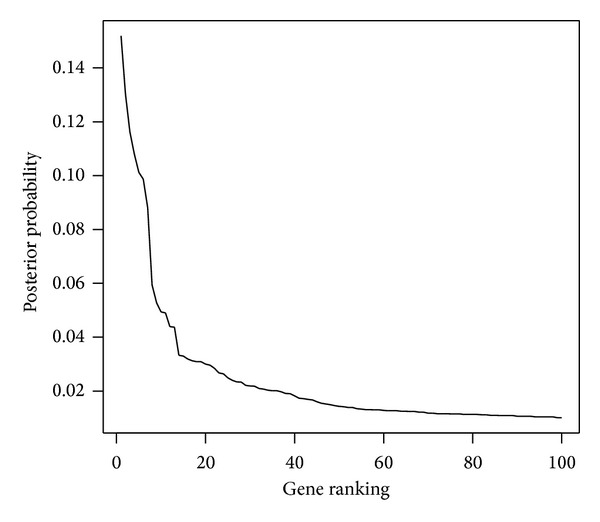
The largest 100 posterior probabilities of the genes for colon cancer study.

The data of the colon cancer study contained 2000 expression levels from 40 tumor and 22 normal colon tissues. These expression levels were first transformed with a base 10 logarithmic function and then standardized to zero mean and unit variance for each gene. We then performed the MCMC sampler fixing the *h* in Σ_*γ*_ at 100 and *π*_*i*_ = Pr(*γ*_*i*_ = 1) = 0.005 for all *i* = 1,…, *p*. We burned in the first 12000 iterations, collected every 30th sample, and obtained 6700 posterior points in total for further analysis. The leading 20 genes with the largest posterior inclusion probabilities were presented in Table 1. This list was compared with the findings in three other studies [38–40] and similar findings were denoted in Table 1. The first 19 genes were identified in at least one of the three studies. For reference, Figure 1 displays the 100 largest posterior probabilities of the 100 corresponding genes.

For classification, we adopted the external leave-one-out cross-validation (LOOCV) procedure to evaluate the performance of classification with the selected genes. The procedures were the following: (i) removing one sample from the training set; (ii) ranking the genes in terms of *t*-statistics using the remaining samples and retaining the top 50 genes as the starting set to reduce computational burden; (iii) selecting the p* most influential genes from the 50 genes based on our Bayesian method; and (iv) using these p* genes to classify the previously removed sample. The procedures were repeated for each sample in the dataset. With different choices of p* like p* = 6, p* = 10, and p* = 14, the error rates were 0.1452, 0.1452, and 0.1129, respectively. The performance of other methods, including SVM [41]; classification tree followed by 1-Nearest-neighbor and LogitBoost with 100 iterations [42]; MAVE-LD [43]; IRWPLS [44]; supervised group Lasso (SGLasso, [45]) and MRMS [46]; and *t*-test for single markers in probit regression was summarized in Table 2. SVM had the smallest error rate, but it apparently included too many genes (1000 in this set). One other method MRMS+SVM+D1 performed better, with one more correct classification, than our proposed procedure when 6 or 10 genes were selected.

### 3.2. Leukemia Study

**Figure 2:**
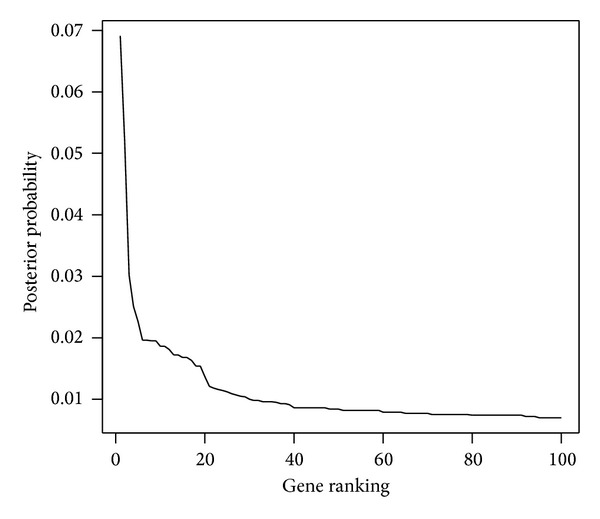
The largest 100 posterior probabilities of the genes for leukemia study.

Next we considered the leukemia study with gene expression levels from 72 tissues including 47 acute lymphoblastic leukemia (ALL) patients and 25 acute myeloid leukemia (AML) subjects. These data contained 38 training and 34 testing samples. The training data contained 27 ALL cases and 11 AML cases, whereas the testing data were with 20 ALL cases and 14 AML cases. As described in other studies [2], the preprocessing steps such as thresholding and filtering were applied first and then followed by a base 10 logarithmic transformation. A total of 3571 genes were left for analysis. Next, we standardized the data across samples, and we ranked these genes by the same MCMC procedures described earlier. The top 20 genes with the largest posterior inclusion probabilities were presented in Table 3, and genes identified by other studies [36, 41, 47, 48] were also noted. For reference, Figure 2 displays the 100 largest posterior probabilities of the 100 corresponding genes.

For the classification procedure, similar to the procedures for colon cancer study, we selected p* most influential genes from a starting set of 50 genes and next used them to examine the testing data. With p* = 6, 10, or 14 genes, only the 61st and 66th observations were misclassified by our procedure. We also compared the results with weighted voting machine [36], MAVE-LD [43], two-step EBM [47], KIGP + PK [48], and *t*-test for single markers with probit regression, as summarized in Table 4. Note that although MAVE-LD and two-step EBM methods performed better than our proposed procedure, both methods used more genes (50 and 512) and yet achieved only one less misclassification. Among this list, our procedure apparently considered a smaller set of genes with a satisfactory performance.

### 3.3. Diffuse Large B-Cell Lymphoma (DLBCL) Study

This study collected 58 samples from DLBCL patients and 19 samples from follicular lymphoma [37]. The original dataset contained 7129 genes. After the preprocessing steps such as thresholding and filtering were applied and a base 10 logarithmic transformation was conducted, a total of 6285 genes were left for analysis. Next, we standardized the data across samples and ranked these genes by the same MCMC procedures described in earlier sections. The error rates for p* = 6, 10, or 14 under LOOCV were 0.0519, 0.0649, and 0.0779, and the accuracy was between 0.92 and 0.95, as listed in Table 5. To achieve a smaller error rate, we considered p* = 5 and obtained a smaller rate 0.0390, the same rate achieved by the hyperbox enclosure (HBE) method [49]. Similar to the discussion in the previous two applications, our proposed model can achieve the same or smaller error rate with a smaller set of genes.

## 4. Conclusion and Discussion

In this Bayesian framework, we considered a mixture of *g*-prior to complete a fully Bayesian analysis for gene selection and cancer classification. Different from other existing methods that treated the *c* as a fixed value, we incorporated its uncertainty by assuming a reference inverse-gamma prior distribution. Earlier studies mentioned this prior, but considered it difficult to derive posterior inference. We therefore outlined the implementation for computation under this model setting for future applications. This approach is more flexible in the process of model building. This model is able to evaluate how influential a gene can be with posterior probabilities that can be used next for variable selection. Such an approach is useful in biomedical interpretations for the selection of relevant genes for disease of interest. When compared with other existing methods, our proposed procedure achieves a better or comparable accurate rate in classification with fewer genes. In the analyses of colon cancer and leukemia studies, we replicate several relevant genes identified by other research groups. The findings have accumulated evidence for further laboratory research.

**Figure 3:**
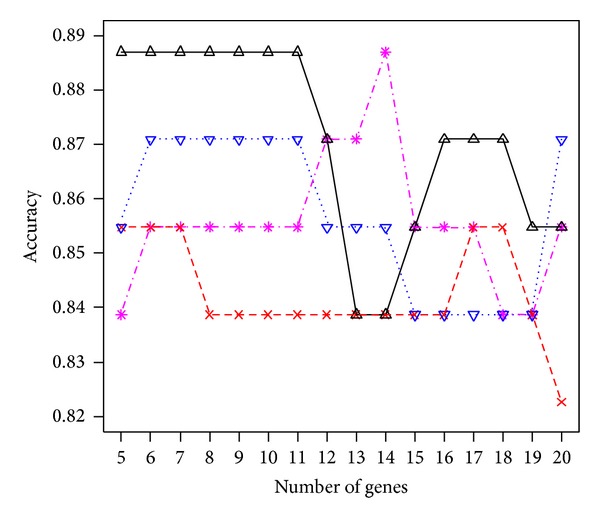
The accuracy of the proposed procedure at different numbers (p* = 5,…, 20) of selected genes with *c* following the generalized *g*-prior (pink line) or fixed at constant 5 (red line), 10 (blue), or 20 (black) for the colon cancer study.

**Figure 4:**
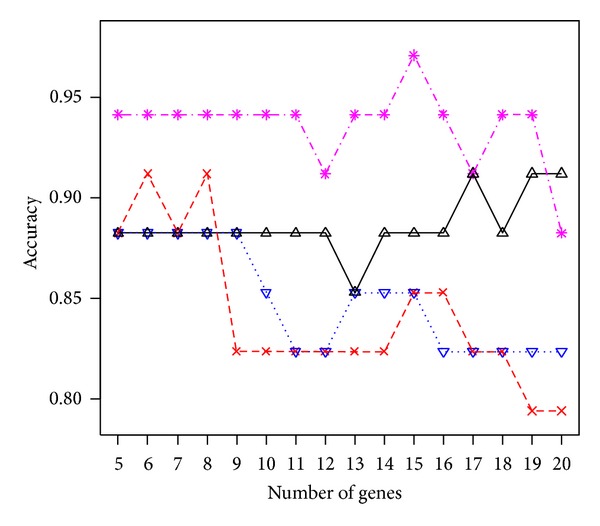
The accuracy of the proposed procedure at different numbers (p* = 5,…, 20) of selected genes with *c* following the generalized *g*-prior (pink line) or fixed at constant 5 (red line), 10 (blue), or 20 (black) for the leukemia study.

In the application section, we listed only the results from p* = 6, 10, and 14 selected genes. Other values for p* have been tried and the performance remains good. For instance, the pink line in Figures 3 and 4 displays the accuracy of the proposed procedure when the number of selected genes p* varies between 5 and 20 for the colon cancer and leukemia study, respectively. For the colon cancer study, the largest accuracy 0.8871 occurs at p* = 14, while other values of p* lead to the accuracy between 0.8387 and 0.8871. These correspond to at least 52 correctly identified subjects out of 62. For the leukemia study, the largest accuracy 0.9706 occurs at p* = 15. Other values of p* all lead to an accuracy larger than 90% except when p* = 20 (accuracy is 0.8824 = 30/34). In addition, we compared the results under the proposed generalized *g*-prior with *c* fixed at a constant. The colored lines in Figures 3 and 4 are for *c* fixed at 5 (red line), 10 (blue), or 20 (black), respectively. Again, results under the prior distribution assumption lead to a higher accuracy with a less number of selected genes. Another issue is related to the choice of the number of genes in the starting set. We have considered 50 in all three applications. This value can certainly be changed. However, the computational complexity increased as the value becomes larger. This cost in computation remains a research topic for future research.

**Figure 5:**
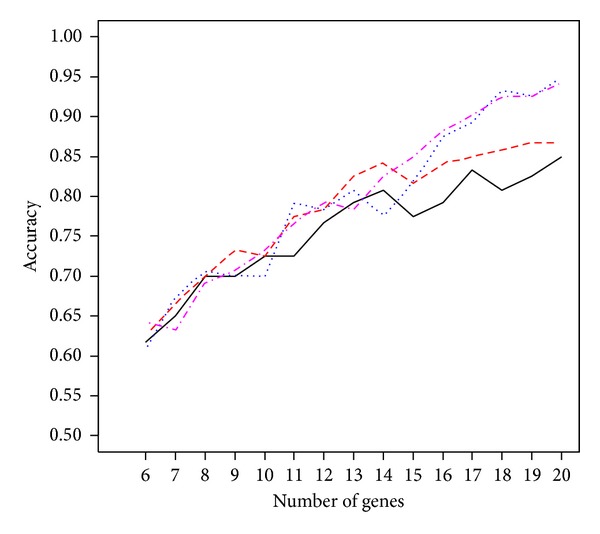
Average accuracy when the number of genes ranges from 1 to 15 under the mixtures of *g*-priors on *c* (pink line), *c* fixed at 5 (black), *c* at 50 (red), and *c* at 500 (blue).

To compare the performance of a stochastic *c* and a constant *c*, we also conducted a small simulation study to investigate the effect of assigning a prior on *c* versus fixing *c* at different constant values. We used the R package penalizedSVM [50, 51] to simulate three data sets; each contains 500 genes with 15 genes associated with the disease. The numbers of training and testing sample were 200 and 40, respectively. We then conducted the gene selection procedures with a prior on *c*, *c* = 5, *c* = 50, and *c* = 500 at p* = 1, 2, …, 15 and recorded the accuracy under each setting.  Figure 5 plots the average accuracy with the pink line standing for the accuracy under the mixtures of *g*-priors on *c*, the black line for *c* = 5, the red line for *c* = 50, and the blue line for *c* = 500. It can be observed that only when *c* is assigned with a very large number like 500, the corresponding accuracy can be slightly better than that under a prior for the uncertainty in *c*. This again supports the use of the mixtures of *g*-priors for a better and robust result.

Here in this paper we have focused on the analysis of binary data. However, the probit regression model can be extended to a multinomial probit model to solve the multiclass problems, and the Bayesian inference can be carried out similarly. Such analysis will involve a larger computational load and further research in this direction is needed. Another point worth mentioning is the inclusion of interactions between genes. Further research can incorporate a power prior into the prior of **γ** [52] or include information on gene-gene network structure [18] to complete the procedure for variable selection.

## Figures and Tables

**Table 1:** The posterior inclusion probability and description of the leading 20 genes for the colon cancer study. Genes identified in other studies were also noted.

Gene	Probability	Description
Z50753	0.1519	*H. sapiens* mRNA for GCAP-II/uroguanylin precursor^abc^
D14812	0.1303	Human mRNA for ORF, complete cds^bc^
H06524	0.1163	Gelsolin precursor, plasma (*Homo sapiens*)^ac^
R87126	0.1081	Myosin heavy chain, nonmuscle (*Gallus gallus*)^abc^
H08393	0.1012	Collagen alpha-2(XI) chain (*Homo sapiens*)^abc^
T62947	0.0987	60S ribosomal protein L24 (*Arabidopsis thaliana*)^abc^
T57882	0.0881	Myosin heavy chain, nonmuscle type A (*Homo sapiens*)^b^
R88740	0.0594	Atp synthase coupling factor 6, mitochondrial precursor (*Homo sapiens*)^bc^
J02854	0.0527	Myosin regulatory light chain 2, smooth muscle isoform (*Homo sapiens*); contains TAR1 repetitive element^ab^
T94579	0.0494	Human chitotriosidase precursor mRNA, complete cds^b^
H64807	0.0490	Placental folate transporter (*Homo sapiens*)^bc^
M59040	0.0439	Human cell adhesion molecule (CD44) mRNA, complete cds^c^
R55310	0.0437	S36390 mitochondrial processing peptidase^c^
M82919	0.0333	Human gamma aminobutyric acid (GABAA) receptor beta-3 subunit mRNA, complete cds^bc^
H20709	0.0330	Myosin light chain alkali, smooth-muscle isoform (*Homo sapiens*)^bc^
T92451	0.0319	Tropomyosin, fibroblast, and epithelial muscle-type (*Homo sapiens*)^a^
R33481	0.0312	Transcription factors ATF-A and ATF-A-DELTA (*Homo sapiens*)^b^
L06175	0.0309	*Homo sapiens* P5-1 mRNA, complete cds
T64012	0.0309	Acetylcholine receptor protein, delta chain precursor (*xenopus laevis*)
H09719	0.0300	Tubulin alpha-6 chain (*Mus musculus*)

^a^Gene also identified in Ben-Dor et al. [38].

^b^Gene also identified in Furlanello et al. [39].

^c^Gene also identified in Chu et al. [40].

**Table 2:** Performance comparison of different procedures with LOOCV for the colon cancer study.

Methods	No. of genes	LOOCV error rate	LOOCV accuracy
Bayesian *g*-prior	6	0.1452 (9/62)	0.8548 (53/62)
Bayesian *g*-prior	10	0.1452 (9/62)	0.8548 (53/62)
Bayesian *g*-prior	14	**0.1129 **(**7/62**)	**0.8871 **(**55/62**)
SVM^a^	1000	**0.0968 **(**6/62**)	**0.9032 **(**56/62**)
Classification tree^b^	200	0.1452 (9/62)	0.8548 (53/62)
1-Nearest-neighbor^b^	25	0.1452 (9/62)	0.8548 (53/62)
LogitBoost, estimated^b^	25	0.1935 (12/62)	0.8065 (50/62)
LogitBoost, 100 iterations^b^	10	0.1452 (9/62)	0.8548 (53/62)
AdaBoost, 100 iterations^b^	10	0.1613 (10/62)	0.8387 (52/62)
MAVE-LD^c^	50	0.1613 (10/62)	0.8387 (52/62)
IRWPLS^d^	20	**0.1129 **(**7/62**)	**0.8871 **(**55/62**)
SGLasso^e^	19	0.1290 (8/62)	0.8710 (54/62)
MRMS + SVM + D1^f^	5	0.1290 (8/62)	0.8710 (54/62)
MRMS + SVM + D2^f^	33	0.1452 (9/62)	0.8548 (53/62)
*t*-test + probit regression	6	0.1452 (9/62)	0.8548 (53/62)
*t*-test + probit regression	10	0.1774 (11/62)	0.8226 (51/62)
*t*-test + probit regression	14	0.2258 (14/62)	0.7742 (48/62)

^a^Proposed by Furey et al. [41].

^b^Proposed by Dettling andBühlmann [42].

^c^Proposed by Antoniadis et al. [43].

^d^Proposed by Ding and Gentleman [44].

^e^Proposed by Ma et al. [45].

^f^Proposed by Maji and Paul [46].

**Table 3:** The posterior inclusion probability and description of the leading 20 genes for the leukemia study. Genes identified in other studies were also noted.

Gene	Probability	Description
X95735	0.0691	Zyxin^abc^
M27891	0.0519	CST3 cystatin C (amyloid angiopathy and cerebral hemorrhage)^abc^
M23197	0.0302	CD33 cD33 antigen (differentiation antigen)^abc^
Y12670	0.0251	LEPR leptin receptor^a^
X85116	0.0226	Epb72 gene exon 1^ab^
D88422	0.0196	CYSTATIN A^bc^
X62654	0.0196	ME491 gene extracted from *H. sapiens* gene for Me491/CD63 antigen^b^
X04085	0.0195	Catalase (EC 1.11.1.6) 5′ank and exon 1 mapping to chromosome 11, band p13 (and joined CDS)^a^
L09209	0.0195	APLP2 amyloid beta (A4) precursor-like protein 2^bc^
HG1612-HT1612	0.0186	Macmarcks^bc^
M16038	0.0186	LYN V-yes-1 Yamaguchi sarcoma viral related oncogene homolog^abc^
U50136	0.0181	Leukotriene C4 synthase (LTC4S) gene^ab^
M55150	0.0172	FAH fumarylacetoacetate^ab^
M92287	0.0172	CCND3 cyclin D3^bc^
M22960	0.0168	PPGB protective protein for beta-galactosidase (galactosialidosis)^bc^
X70297	0.0168	CHRNA7 cholinergic receptor, nicotinic, and alpha polypeptide 7^b^
X51521	0.0163	VIL2 Villin 2 (ezrin)^b^
M63138	0.0154	CTSD cathepsin D (lysosomal aspartyl protease)^ab^
M27783	0.0154	ELA2 elastase 2, neutrophil^c^
U81554	0.0137	CaM kinase II isoform mRNA

^a^Gene also identified in Golub et al. [36].

^b^Gene also identified in Ben-Dor et al. [38].

^c^Gene also identified in in Lee et al. [22].

**Table 4:** Performance comparison of different procedures for the leukemia study.

Methods	No. of genes	Testing error rate	Testing accuracy
Bayesian *g*-prior	6	**0.0588 **(**2/34**)	**0.9412 **(**32/34**)
Bayesian *g*-prior	10	0.0588 (2/34)	0.9412 (32/34)
Bayesian *g*-prior	14	0.0588 (2/34)	0.9412 (32/34)
Weighted voting machine^a^	50	0.1471 (5/34)	0.8529 (29/34)
MAVE-LD^b^	50	**0.0294 **(**1/34**)	**0.9706 **(**33/34**)
Two-step EBM^c^	32	0.1471 (5/34)	0.8529 (29/34)
Two-step EBM^c^	256	0.0588 (2/34)	0.9412 (32/34)
Two-step EBM^c^	512	**0.0294 **(**1/34**)	**0.9706 **(**33/34**)
KIGP + PK^d^	20	0.0588 (2/34)	0.9412 (32/34)
*t*-test + probit regression	6	0.1765 (6/34)	0.8235 (28/34)
*t*-test + probit regression	10	0.0882 (3/34)	0.9118 (31/34)
*t*-test + probit regression	14	0.1176 (4/34)	0.8824 (30/34)

^a^Proposed by Gloub et al. [36].

^b^Proposed by Antoniadis et al. [43].

^c^Proposed by Ji et al. [47].

^d^Proposed by Zhao and Cheung [48].

**Table 5:** Performance comparison of different procedures with LOOCV for the colon cancer study.

Methods	No. of genes	LOOCV error rate	LOOCV accuracy
Bayesian *g*-prior	5	0.0390 (3/77)	0.9610 (74/77)
Bayesian *g*-prior	6	0.0519 (4/77)	0.9481 (73/77)
Bayesian *g*-prior	10	0.0649 (5/77)	0.9351 (72/77)
Bayesian *g*-prior	14	0.0779 (6/77)	0.9221 (71/77)
Bayesian *g*-prior	20	0.0779 (6/77)	0.9221 (71/77)
HBE	6	0.0390 (3/77)	0.9610 (74/77)
*t*-test + probit regression	6	0.1169 (9/77)	0.8831 (68/77)
*t*-test + probit regression	10	0.1558 (12/77)	0.8442 (65/77)
*t*-test + probit regression	14	0.2208 (17/77)	0.7792 (60/77)
